# Non-invasively predicting differentiation of pancreatic cancer through comparative serum metabonomic profiling

**DOI:** 10.1186/s12885-017-3703-9

**Published:** 2017-11-02

**Authors:** Shi Wen, Bohan Zhan, Jianghua Feng, Weize Hu, Xianchao Lin, Jianxi Bai, Heguang Huang

**Affiliations:** 1 Department of General Surgery, Fujian Medical University Union Hospital, Fuzhou, 350001 China; 2 Department of Electronic Science, Fujian Provincial Key Laboratory of Plasma and Magnetic Resonance, Xiamen University, Xiamen, 361005 China

**Keywords:** Pancreatic ductal adenocarcinoma, Nuclear magnetic resonance, Metabonomics, Tumor differentiation

## Abstract

**Background:**

The differentiation of pancreatic ductal adenocarcinoma (PDAC) could be associated with prognosis and may influence the choices of clinical management. No applicable methods could reliably predict the tumor differentiation preoperatively. Thus, the aim of this study was to compare the metabonomic profiling of pancreatic ductal adenocarcinoma with different differentiations and assess the feasibility of predicting tumor differentiations through metabonomic strategy based on nuclear magnetic resonance spectroscopy.

**Methods:**

By implanting pancreatic cancer cell strains Panc-1, Bxpc-3 and SW1990 in nude mice in situ, we successfully established the orthotopic xenograft models of PDAC with different differentiations. The metabonomic profiling of serum from different PDAC was achieved and analyzed by using ^1^H nuclear magnetic resonance (NMR) spectroscopy combined with the multivariate statistical analysis. Then, the differential metabolites acquired were used for enrichment analysis of metabolic pathways to get a deep insight.

**Results:**

An obvious metabonomic difference was demonstrated between all groups and the pattern recognition models were established successfully. The higher concentrations of amino acids, glycolytic and glutaminolytic participators in SW1990 and choline-contain metabolites in Panc-1 relative to other PDAC cells were demonstrated, which may be served as potential indicators for tumor differentiation. The metabolic pathways and differential metabolites identified in current study may be associated with specific pathways such as serine-glycine-one-carbon and glutaminolytic pathways, which can regulate tumorous proliferation and epigenetic regulation.

**Conclusion:**

The NMR-based metabonomic strategy may be served as a non-invasive detection method for predicting tumor differentiation preoperatively.

## Background

Pancreatic ductal adenocarcinoma (PDAC) is one of the most malignant tumors with an extremely poor prognosis. Only about 7% of patients can be survived in 5 years, making PDAC the fourth leading cause of death among tumors [1]. Many risk factors have been correlated with prognosis, including tumor size [2, 3], lymph node metastasis [3, 4], nerve plexus invasion [5, 6], vascular invasion [6, 7], tumor differentiation [2, 3, 8], surgical margin status [3, 9] and specific molecular prognostic factors [10, 11]. Thereinto, poorly differentiated/high grade tumors are closely associated with poor outcome of the patients [12]. Furthermore, previous researches also linked tumor histological grading to an increased risk of early death within 1 year [13, 14]. As an important component of early mortality risk score, tumor differentiation can help to assessing short-term tumor-related mortality [14, 15]. Given the important role of tumor differentiation in PDAC management, increased interest in preoperative tumor differentiation assessment were emerged in order to identify high-risk patients, which can benefit the most from neoadjuvant treatment [13, 16–19], even over than upfront surgery [20, 21]. Thus, notarizing differentiation of tumors preoperatively can provide constructive information for prognostic evaluation and management of PDAC [22].

Conventionally, the preoperative assessments of tumor differentiation were conducted by tissue histological observations derived from fine needle aspiration. This method has been realized to be an effective way to grade the pancreatic neuroendocrine tumors and intraductal papillary mucinous neoplasms [23, 24]. However, this technique is highly invasive for many patients and the achievable samples are too limited to give a reliable histological grading, making this technique still being far away from application in clinical PDAC differentiation assessment [19]. Thus, it would be of great importance to develop an easily acceptable and reliable method to assess the differentiation of PDAC preoperatively.

Nuclear magnetic resonance (NMR) spectroscopy-based metabonomic technique is a promising diagnostic tool with the advantages of high sensitivity, non-invasion and high throughput. This technique can analyze the disease-related metabonomic differences occurred in various types of biosamples (etc. tissues, body fluids and cells) to identify differential metabolites and further biomarkers contributed to establishment of recognition models for diagnosis. At present, NMR-based diagnostic strategy has demonstrated a favorable clinical performance in many diseases [25–31]. Particularly noticeable, magnetic resonance spectroscopy have also been recommended for diagnosis of brain, prostate and breast cancer in European cancer conference [29]. In addition, by using NMR-based methods, many reports on detecting PDAC *in vivo* or *in vitro* have showed an encouraging result to distinguish PDAC from not only the normal but also other benign lesions [32–35]. Therefore, in present study, we used ^1^H NMR spectroscopy to analyze serum metabonomes from PDAC mice models established by implantations of Panc-1, BxPC-3 and SW1990 (being poor, poor to moderate and moderate to well differentiated [36–39], respectively) cell strains on pancreas, thus, to assess the feasibility of this strategy in predicting the differentiation of tumor.

## Methods

### Cell culture and animals feeding

PDAC cell strains (Panc-1, BxPC-3 and SW1990, Catalog NO. SCSP-535, TCHu 12 and TCHu 201) were obtained from Shanghai Institute of Cell Biology, Chinese Academy of Sciences (Shanghai, China) authenticated with short tandem repeat test and mycoplasma culture. At the circumstance of 5% CO_2_ and 37 °C, these strains were incubated in dulbecco’s modified eagle medium (DMEM, Gibco, Thermo Fisher Scientific Inc., USA) added with 10% fetal bovine serum (Gibco) in cell incubator (3110, Thermo Scientific). Then, cells were digested by 0.125% trypsinogen (Life Technologies, Grand Island, NY, USA) for the passage with the ratio of 1:2-4 every 2-3 days. BALB/c nude mice (male, 4 weeks, weighing 18-20 g), purchased from Shanghai Slac laboratory animals Co., Ltd. (NO: SCXK (HU) 2012-0002), were bred in Fujian Medical University Animals Centre (Fuzhou, china) with a standard SPF-grade laboratory conditions.

### Establishment of animal models

This experimental protocol was in accordance with the principles of National Institutes of Health guide for the care and use of laboratory animals and approved by Ethical Committee of Fujian Medical University. Three PDAC cell strains in the exponential phase were digested with 0.125% trypsinogen, washed by phosphate buffered saline (PBS) for three times, then collected and resuspended in PBS (1 × 10^7^ cells per milliliter). After skin degerming, the cell suspension liquids were subcutaneously injected into the axilla of mice (one cell strain each mouse), followed by a month of normal feeding. The tumors with a size of 5 to 10 mm in diameter generated in the injected positions of mice. Consequently, the mice were executed by a mercy killing, and the tumor tissues of Panc-1, BxPC-3 and SW1990 were carefully collected and divided into pieces of 1 mm^3^ for implantation *in situ*.

Forty-five mice were randomly divided into 3 groups using random number table. Before surgery, all mice have a 12-h fasting without drink-deprivation. A 2-cm horizontal incision was made on the middle of abdominal wall to expose the pancreas. One piece of tumors was placed on the body or tail of pancreas and fixed with biogum (BaiYun medical glue Co., Ltd., Guangzhou, China), followed by carefully organ restoration and suture. Three groups were dealt with tumor tissues of Panc-1, BxPC-3 and SW1990, respectively (*n* = 15 for each).

### Tissues samples collection and preparation

Thirty days after surgeries, 1 mL of blood from each group was collected by aortic puncture under continuous airway anesthesia of isoflurane (Jiupai pharmaceutical Co., Ltd., Shijiazhuang, China) and stored in clear 1.5-mL Eppendorf tubes. After standing for 30 min, the blood went through a 10-min centrifugation at 10,000 g and 4 °C. The supernate was collected and immediately frozen by liquid nitrogen and stored at −80 °C. For the detection of ^1^H NMR spectroscopy, 400 μL of serum were melted on the surface of ice, and then mixed with 200 μL of 90 mM deuterated phosphate buffer (NaH_2_PO_4_ and K_2_HPO_4_, pH 7.4). The mixture of serum and buffer were centrifuged again, and finally, 550 μL of the supernate was moved into 5-mm NMR tubes (ST500, NORELL, Inc., Morganton, North Carolina, USA).

### Detection of 1H NMR spectroscopy and preprocessing

The ^1^H NMR spectroscopy of serum samples were performed on a Varian NMR system (Agilent Technologies Co, Palo Alto, California, USA) with a 500.13 MHz of proton frequency at the temperature of 298 K. For each sample, a water-suppressed CPMG (Carr-Purcell-Meiboom-Gill) spin-echo pulse sequence (RD-90°-(τ-180°-τ)_n_-ACQ) was used to acquire the NMR spectrum. Herein, a total of 64 scans with a spectral width of 6 KHz and a data point of 12 K were accumulated for all spectra. Spin-echo loop time (2nτ) of 70 ms was applied with a relaxation delay of 2.0 s. The NMR spectra were processed by using MestReNova (V9.0.1, Mestrelab Research S. L., Spain). In order to increase the signal-to-noise ratio, all free induction decays were multiplied by an exponential weighting function equivalent to a 1 Hz line-broadening and subsequently disposed with Fourier transformation. To make the spectra more comparable, the manual phase rectifications and baseline corrections were conducted by using MestReNova. The chemical shifts were referenced to the double-peak of endogenic lactate at δ1.33 for metabolites identification. Automatically, the spectral regions δ9.0-0.5 of the processed NMR spectra were segmented into scatter integral regions of 0.002 ppm with a removal of spectral region δ6.40-5.50 and δ5.19-4.36 to eliminate the impacts of residual water signal and urea signal, respectively. Finally, for each spectrum, the integrated data were normalized to the total sum of the spectrum in favour of multivariate statistical analysis.

### Multivariate statistical analysis

The multivariate statistical analysis, including principal component analysis (PCA), partial least squares discriminant analysis (PLS-DA) and orthogonal partial least squares discriminant analysis (OPLS-DA), were performed in SIMCA-P^+^ (V14.0 Umetrics, Sweden) to analyze the metabonomic differences between three PDAC groups. PCA, performed in the approach of mean-centered scaling, could simplify the normalized date into several components, which can roughly evaluate the clusters distributions and identify the existence of outlines. PLS-DA and OPLS-DA, which can be classified as supervised multivariate statistical analysis, were conducted in the approach of parato-scaling approach for better extraction and maximization of the metabonomic differences between PDAC groups. Furthermore, the OPLS-DA models coefficients, which were back-calculated from the coefficients, incorporated with the weight of the variables, and then to be plotted with color-coded coefficients to enhance interpretability of the models. As a result, the metabolites responsible for the metabonomic differences between groups can be extracted from the corresponding color-coded loading plots and displayed visually. By assistance of MATLAB (V7.1, the Mathworks Inc., Natick, USA), the color-coded coefficient loading plots were drew and color-coded according to the absolute value of coefficient. That meant, in the loading plots, a warm-toned color (i.e. red) represents for the metabolites being positive or negative significant in distinguishing different groups while a cool-toned color (i.e. blue) corresponds to the metabolites not being significant in discriminations. Moreover, to screen out differential metabolites, the cutoff value of correlation coefficients (|r| > 0.576) was determined according to the statistical significance of the Pearson correlation coefficient test at the level of *P* < 0.05 and *df* (degree of freedom) =10. In order to assess the quality and validity of models, the 10-fold cross validation and response permutation testing (*n* = 200) were performed and the corresponding parameters R^2^ and Q^2^ in the permutated plots presented the degree of model fitting and the potentially predictive ability of models, respectively.

#### The metabolic pathways and interactions analysis

The differential metabolites derived from multivariate statistical analysis were further analyzed for the metabolic pathways by using KEGG (www.genome.jp/kegg) and MBROLE 1.0 (http://csbg.cnb.csic.es/mbrole/) [40, 41].

## Results

### NMR spectral profiles of serum samples from Panc-1, BxPC-3, SW1990 groups

**Fig. 1:**
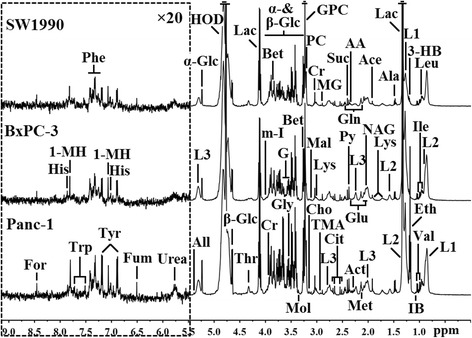
Representative 500 MHz ^1^H CPMG NMR spectra of serum samples from pancreatic cancer mice induced by the different differentiated cells. The spectral regions of δ5.5-9.0 (in the dashed box) were magnified 20 times compared with the regions of δ0.0-5.5 for the purpose of clarity. The abbreviations for peak assignments were noted in Table 1

After visual confirmation for tumorgenesis, 12, 13, and 11 serum samples from Panc-1, BxPC-3 and SW1990 groups were included for the detections with ^1^H NMR spectroscopy, respectively. Typical one-dimensional 500-MHz ^1^H NMR spectra of serum samples from models induced by the different differentiated PDAC cells are presented in Fig. 1, which provided an integrated overview of all metabolites. Forty-seven metabolites were identified from the NMR spectra (Table 1) based on the relative literatures and public databases [42, 43]. A certain degree of metabolic differences could be noticed between different PDAC groups visually such as ethanol and phosphocholine. But considering the high similarity of spectra, the metabonomic information acquired was quite limited and the multivariate statistic analysis will help to extract the detailed information.

**Table 1:** The metabolites assignments from NMR spectra of serum from PDAC mice^a^

Abbreviation	Metabolites	^1^H chemical shift(multiplicity)^b^
1-MH	1-Methylhistidine	7.06(s), 7.78(s)
3-HB	3-Hydroxybutyrate	1.20(d), 2.31(dd), 2.40(m), 4.16(m)
Ace	Acetate	1.92(s)
AA	Acetoacetate	2.28(s)
Act	Acetone	2.24(s)
Ala	Alanine	1.48(d)
All	Allantoin	5.39(s)
Bet	Betaine	3.27(s), 3.90(s)
Cho	Choline	3.20(s)
Cit	Citrate	2.53(d), 2.67(d)
Cr	Creatine	3.04(s), 3.93(s)
Eth	Ethanol	1.18(t), 3.61(q)
For	Formate	8.46(s)
Fum	Fumarate	6.52(s)
Glu	Glutamate	2.08(m), 2.11(m), 2.35(m), 3.75(t)
Gln	Glutamine	2.14(m), 2.45(m), 3.75(t)
G	Glycerol	3.55(m), 3.66(dd), 3.78(m)
GPC	Glycerolphosphocholine	3.23(s), 4.33(m)
Gly	Glycine	3.56(s)
His	Histidine	7.08(s), 7.82(s)
HOD	Residual water signal	4.76(br)
IB	Isobutyrate	1.07(d)
Ile	Isoleucine	0.94(t), 1.01(d)
L1	LDL	0.86(br), 1.28(br)
L2	VLDL	0.89(br), 1.30(br), 1.58(br)
L3	Unsaturated fatty acid	2.04(br), 2.24(br), 2.76(br), 5.31(br)
Lac	Lactate	1.33(d), 4.11(q)
Leu	Leucine	0.96(d)
Lys	Lysine	1.46(m), 1.73(m), 1.91(m), 3.03(m), 3.76(t)
Mal	Malonate	3.11(s)
Met	Methionine	2.14(s), 2.63(t)
MG	Methylguanidine	2.83(s), 3.36(s)
Mol	Methanol	3.36(s)
m-I	*myo*-Inositol	3.52(dd), 3.61(dd), 4.07(m)
NAG	N-acetyl glycoprotein	2.03(s)
Phe	Phenylalanine	7.32(d), 7.37(t), 7.42(dd)
PC	Phosphocholine	3.21(s)
Py	Pyruvate	2.37(s)
Suc	Succinate	2.40(s)
Thr	Threonine	1.33(d), 4.26(m)
TMA	Trimethylamine	2.89(s)
Trp	Tryptophan	7.27(m), 7.30(s), 7.54(d), 7.73(d)
Tyr	Tyrosine	6.90(d), 7.19(d)
Urea	Urea	5.80(br)
Val	Valine	0.99(d), 1.04(d)
α-Glc	α-Glucose	3.42(t), 3.54(dd), 3.71(t), 3.73(m), 3.84(m), 5.24(d)
β-Glc	β-Glucose	3.24(ddb), 3.41(t), 3.46(m), 3.49(t), 3.90(dd), 4.65(d)

^a^ *PDAC* pancreatic ductal adenocarcinoma

^b^multiplicity:s, singlet; d, doublet; t, triplet; q, quartet; dd, doublets; m, multiplet; br, broad resonance

### Metabonomic characteristics of serum from the PDAC groups

**Fig. 2:**
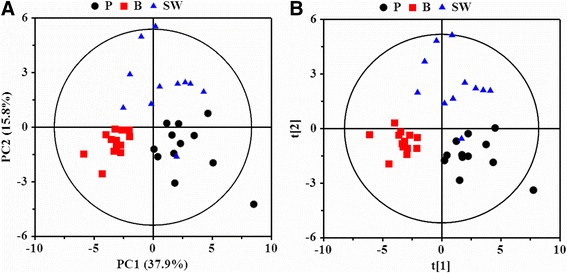
The PCA (**a**) and PLS-DA (**b**) scores plots based on ^1^H NMR data of serums from PDAC groups. P, Panc-1; B, BxPC-3; SW, SW1990

To show an overview of ^1^H NMR data collected from the serum of Panc-1, BxPC-3, and SW1990 groups, the PCA and PLS-DA were performed. The PCA scores plot showed a certain degree of separated trends between the three PDAC groups (Fig. 2a) though a little overlap or dispersity was demonstrated, indicating their obvious metabonomic differences. In further, a greater discrimination in cluster distributions of Panc-1, Bxpc-3 and SW1990 could be observed visually in PLS-DA scores plot (Fig. 2b), demonstrating a significant differences with each other.

**Fig. 3:**
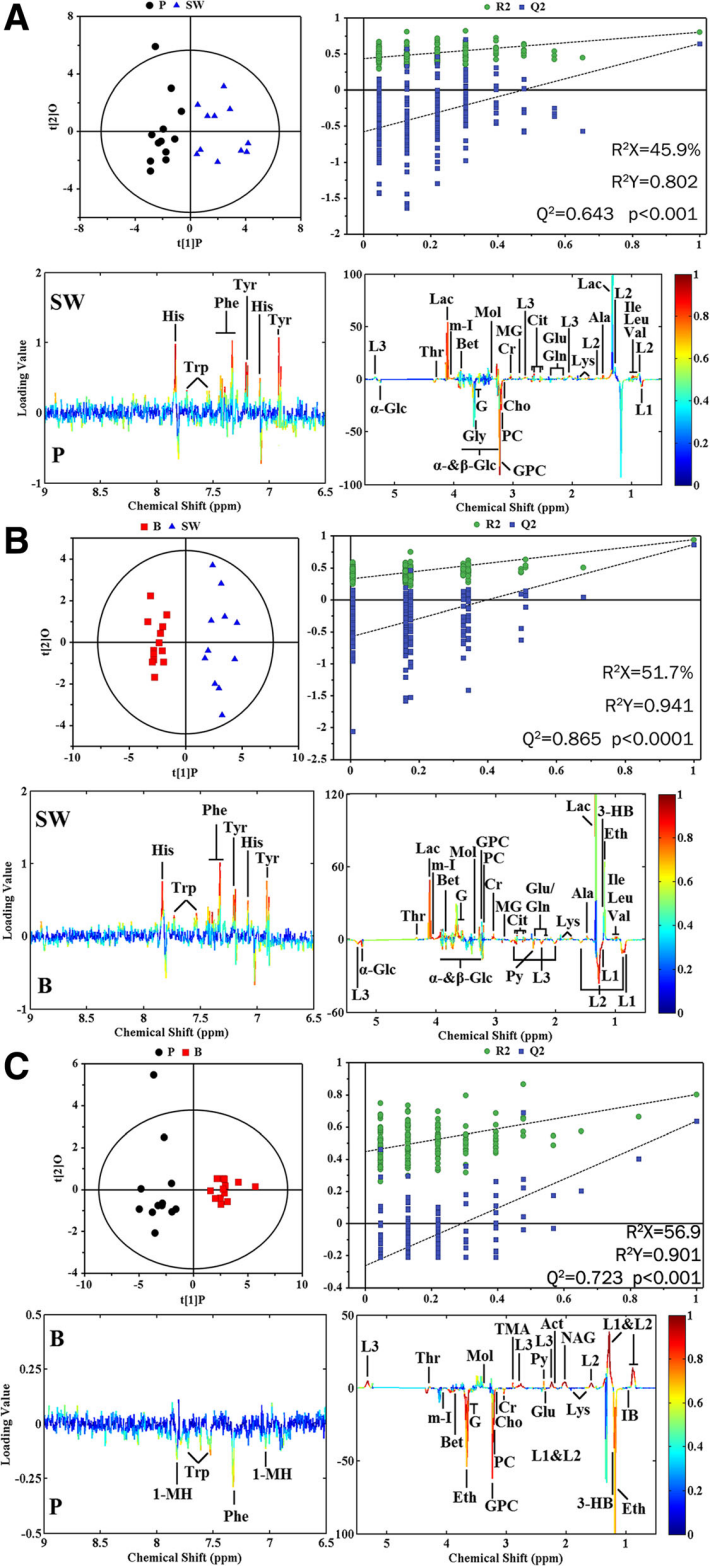
OPLS-DA scores plots (upper left panels) and plots of permutation tests (*n* = 200) (upper right panels) derived from ^1^H NMR spectra of serum samples and corresponding coefficient loading plots (bottom panels) from the pair-wise comparisons between Panc-1, Bxpc-3 and SW1990 groups. **a**. Panc-1 vs SW1990, **b**. BxPC-3 vs SW1990, **c**. Panc-1 vs BxPC-3. The color map shows the significance of metabolites variations between the two classes. Keys of the assignments were shown in Table 1. P, Panc-1; B, BxPC-3; SW, SW1990

To get deep insight into the metabolites responsible for the metabonomic alterations occurred in three PDAC groups, pair-wise comparisons were conducted by using the PLS-DA combined with orthogonal projection (OPLS-DA). The pronounced separations were demonstrated in OPLS-DA scores plots (Fig. 3 upper left panels) and the metabolites corresponding to the metabolic difference were marked in loading plots (Fig. 3 bottom panels). The summarized dominant metabolites, based on the cutoff value of correlation coefficient (|r| > 0.576), and the correlation coefficients were listed in detail based on their biochemical types (Table 2). Overall, the levels of metabolites belonged to glycolysis and glutaminolysis, alcohols and amino acids were lower in SW1990 group while the high concentrations of choline and its derivatives were noticeable in Panc-1 group. The favorable fit and prediction parameters (R^2^ and Q^2^) of the OPLS-DA models and the corresponding permutation test and probability (*p*-value) via CV-ANOVA also confirmed the strong predictive ability of the models to guarantee a reliable identification of characteristic metabolites.

**Table 2:** OPLS-DA coefficients of metabolites in different pair-comparisons derived from NMR-data

Metabolites	r^a^		
	BxPC-3 vs SW1990	Panc-1 vs SW1990	Panc-1 vs BxPC-3
Glycolysis and glutaminolysis			
α-Glucose	−0.788	−0.631	–
β-Glucose	−0.735	−0.842	–
Citrate	0.817	0.921	–
Glutamate	0.808	0.747	−0.789
Glutamine	0.767	0.856	–
Lactate	0.906	0.905	–
pyruvate	−0.880	–	0.793
Succinate	–	–	–
*Carboxylic acids and derivatives*			
Acetate	–	–	–
Formate	–	–	–
Fumarate	–	–	–
Isobutyrate	–	–	−0.709
Malonate	–	–	−0.648
*Alcohols*			
Ethanol	0.879	–	−0.804
Methanol	0.702	0.760	0.667
myo-Inositol	0.889	0.817	−0.877
Glycerol	0.935	0.784	−0.916
Lipid			
LDL	−0.899	−0.847	0.912
VLDL	−0.774	0.720	0.921
Unsaturated fatty acid	−0.899	−0.847	0.912
*ketone body*			
3-Hydroxybutyrate	0.747	–	−0.636
Acetoacetate	–	–	–
Acetone	−0.760	–	0.912
*Choline and derivatives*			
Choline	–	−0.836	−0.841
Glycerolphosphocholine	0.671	−0.912	−0.894
Phosphocholine	0.736	−0.832	−0.892
Amino acid			
*Non-essential amino acid*			
1-methylhistidine	–	–	−0.651
Alanine	0.750	0.778	–
Betaine	0.812	0.834	−0.769
Creatine	0.930	0.826	−0.849
Glycine	0.871	0.674	–
Histidine	0.776	0.602	–
Tyrosine	0.832	0.859	–
*Essential amino acid*			
Isoleucine	0.749	0.795	–
Leucine	0.707	0.775	–
Lysine	0.886	0.822	−0.780
Methionine	–	0.645	–
Phenylalanine	0.878	0.813	−0.642
Threonine	0.630	0.794	0.730
Tryptophan	0.846	0.847	−0.673
Valine	0.839	0.858	–
Others			
Methylguanidine	0.650	0.732	–
Allantoin	−0.687	–	–
N-acetyl glycoprotein	–	0.661	0.914
Trimethylamine	−0.855	0.750	0.782

^a^Correlation coefficients, positive and negative signs indicate positive and negative correlation in the concentrations. |r| > 0.576 was the cutoff value for significance based on discrimination significance of *p* = 0.05 and df = 10. “-” means |r| < 0.576

### The biochemical pathways related with the metabonomic difference between PDAC groups

**Fig. 4:**
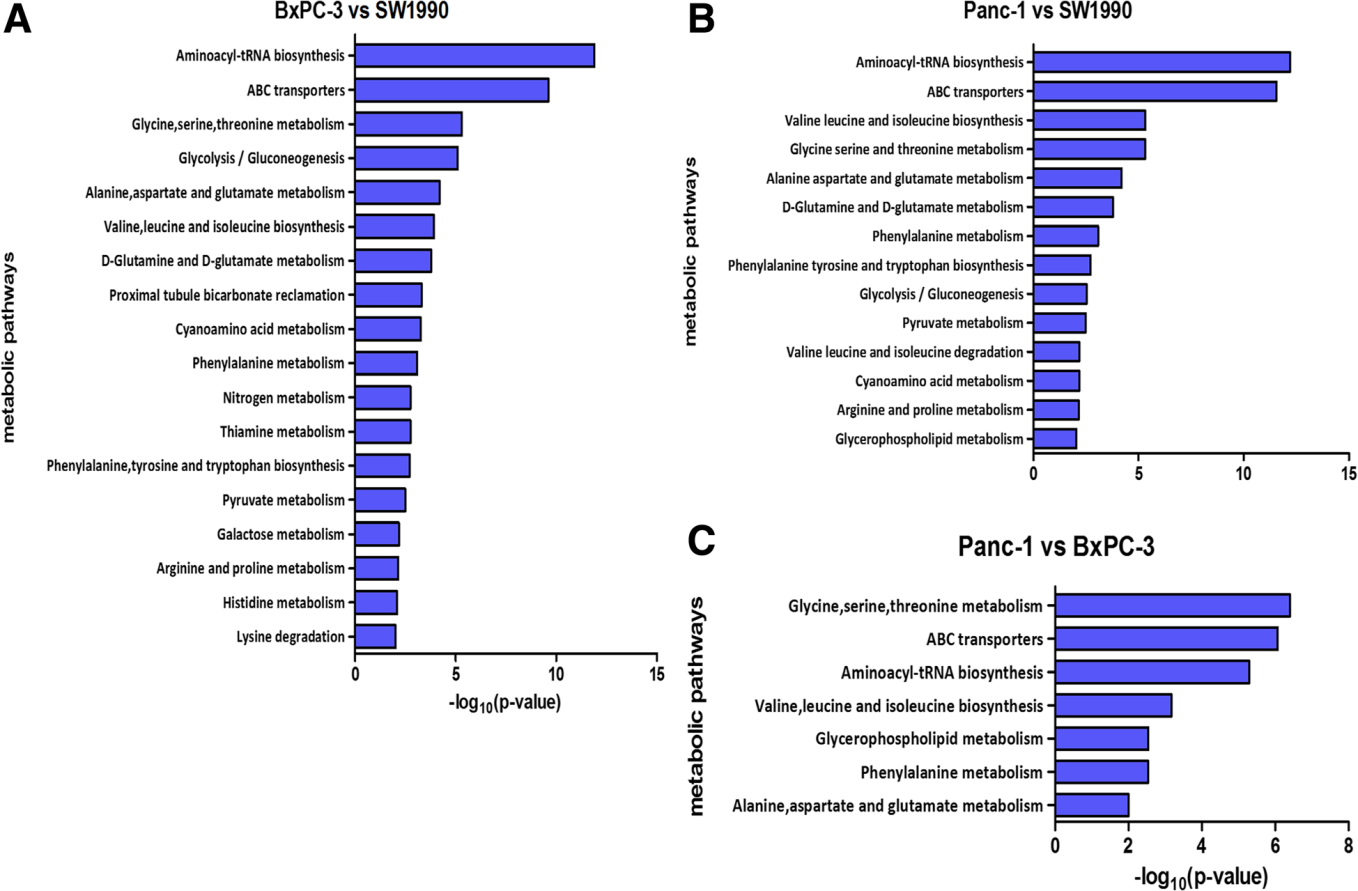
The corresponding pathways drived from the differential metabolites from different pair-comparisons. **a** BxPC-3 vs SW1990, **b** Panc-1 vs SW1990, **c** Panc-1 vs BxPC-3. This pathway analysis was performed in MBROLE online services based on KEGG database. The pathways with *P*-value <0.01(y-axes) and corresponding *P*-values (x-axes) in different pair-comparison were identified and listed

For better understanding of the bioinformation contained in discriminatory metabolites, the biochemical pathways were identified based on the differential metabolites derived from OPLS-DA of pair-comparisons and those with *p*-value less than 0.01 were demonstrated on Fig. 4. The *p*-value for pathway identification were calculated automatically by the MBROLE [40].

In the analysis to compare SW1990 with Bxpc-3, the numerous amino acid-related pathways were noticeable, including metabolism of essential and non-essential amino acids, the biosynthesis of aminoacyl-tRNA and ABC transporters. In addition, the pathways related with glycolysis involving pyruvate, galactose, glutamine and glutamate were also identified as differential features to distinguish the Bxpc-3 from the SW1990. Meanwhile, except the pathways of lysine, histidine and thiamine metabolisms, most pathways involved in Bxpc-3 vs SW1990 were also identified in the comparison between Panc-1 and Sw1990. In addition, the pathways of glycerophospholipid metabolism and the degradation of valine, leucine and isoleucine were also identified to be a signature contributed to distinguish Panc-1 from SW1990. In term of metabolic diversity between Panc-1 and BxPC-3, the metabolic discrimination seems to be quite limited where only a few pathways related with amino acids and glycerophospholipid metabolism were identified.

## Discussion

In this study, we tried to evaluate the potential value of non-targeted NMR strategy to predict the tumor differentiation. Since many factors (e.g., drugs, operations) could influence the metabonomic characteristics of serum from patients. We chose three PDAC strains, Panc-1, BxPC-3 and SW1990 which can form tumors *in vivo* with typical histopathologic characters from poor, poor to moderate and moderate to well differentiation respectively [36–39] to establish PDAC models for research. By using animal models, the interference factors can be furthest eliminated. It is beneficial to purify serum metabonomic alteration caused by tumor with different differentiation and also specify the association between tumor differentiation and serum metabonomes. To amplify the metabolic difference between the tumors in different differentiations, all groups were compared directly. Given most of clinical patients were diagnosed with moderately differentiated PDAC and the significant clinical value for the identification of tumors in poor differentiation, we focus on the metabonomic difference between SW1990 and other two strains.

### Comparative low levels of lactate, glutamate and glutamine indicate a poor differentiation

In present study, we found that the high concentration of citrate, lactate, glutamate and glutamine can help to distinguish the SW1990 from Panc-1 and Bxpc-3. Being well known, the tumor metabolic reprogramming has been validated to be the cornerstone for malignant transformation and one common composition in this process is the aerobic glycolysis (Warburg effect). Through the aerobic glycolysis, rather than tricarboxylic acid (TCA) cycle, the tumor cells derive the predominant ATP/energy and generate extensive lactate from pyruvate to result in environmental acidosis which promote the spreading of the tumor cells [44]. Meanwhile, the lactate generated from hypoxic PDAC can be taken up by normoxic PDAC cells nearby as fuel to maintain proliferation, creating a phenomenon called tumor symbiosis [45]. Thus, the tumor metabolic impact upon the level of lactate in peripheral circulation may be determined by the dynamic balance of release and uptake of lactate around tumor microenvironment. Our outcome indicates that the tumor with a poorer differentiation could induce a lower concentration of lactate in serum relative to that with a better differentiation, which may be due to a stronger ability of lactate recirculation. It’s also implied by inconsistent variation trends of lactate in serum reported by previous studies [46, 47]. In addition, due to the breakdown of TCA cycle, glutaminolysis is enhanced in PDAC cells to generate TCA intermediates (e.g. malate, oxaloacetate and citrate) which is called anaplerosis reaction, and subsequently served as building blocks for synthesis of lipid and non-essential amino acids [48]. Besides, glutamine can also act as fuel to support energy metabolism through aspartate, oxaloacetate and pyruvate transformation process, thus promoting growth of pancreatic cancer via *Kras*-regulated metabolic pathway [49]. Therefore, the significantly low levels of glutamine, glutamate and citrate may indicate that the tumor with poorer differentiation may provide a more dramatic glutaminolysis and deprive more glutamine and glutamate from peripheral circulation.

### Comparative low levels of amino acids in serum imply poor differentiation

Likewise, the higher concentrations of amino acids could also contribute to the distinguishing of the SW1990 from Panc-1 and Bxpc-3, which could serve as key participants in the cancer metabolism reprogramming. Under the influence of the abnormal expression of oncogenes and tumor suppress genes, the anabolic metabolism and transport of amino acid were tremendously enhanced for rapid proliferation of cancer cells. To provide required nutrients for cancer growth, the catabolic metabolism of whole-body tissue would be enhanced, leading to an increased circulating amino acids at the early stage of PDAC [50]. But the catabolic metabolism cannot maintain in a high level for a long time and end in a severe nutritional imbalance called cachexia, thus creating a decrease of amino acids in serum at last. In this process, L-type amino-acid transporter 1 (LAT-1), the most important transporter of neutral amino acids, plays a key role in internalized transportation of essential amino acids (EAAs) in PDAC. As previous reports demonstrated, the overexpression of LAT-1 can promote cancer growth via mammalian target-of-rapamycin (mTOR) and serve as a prognostic factor in PDAC [51, 52]. Thus, the higher concentration of EAAs in SW1990 group than in Panc-1 and BxPC-3 group indicates that the tumors with poor differentiation may have a higher expression of LAT1 and nutritional stress from rapid proliferation, which can associated with poor prognosis.

With regard to the non-essential amino acids (NEAAs), several pathways were involved to enhance their biosynthesis and utilization for cell proliferation. As noted above, the accumulated glycolysis intermediates could also promote the biosynthesis of glycine, serine and threonine through 3-phospho-D-glycerate pathway. In addition, the increased glutaminolysis provides numerous substrates (e.g. isocitrate, malate, alpha-ketoglutaric acid) not only to supply the lipids synthesis but also to promote the biosynthesis of alanine and aspartate. Besides being used as building blocks and fuels for cell proliferation, NEAAs have been indicated to bridge the interplay metabolism and epigenetics, thus serve as programmed switch for cell differentiation [53]. For instance, several NEAAs including glycine could be associated with gene signatures of cell proliferation and *Myc* target activation through the serine-glycine-one-carbon pathway (SGOC pathway), which contribute significantly to energy generation and biosynthesis of NADPH and purine [54]. In addition, the mTOR-dependent induction of SGOC pathways can also lead to DNA methylation and tumorigenesis under the cooperatively oncogenic function of the loss of liver kinase B1 and activation of Kras, which highly involved in epigenetics [55]. Thus, NEAAs are highly associated with genesis, progression and epigenetics, and their relative concentration in serum may be indicators for the differentiation of PDAC.

### Relative high concentration metabolites of choline metabolism may imply a poor differentiation

Impressively, the high correlation coefficient of choline groups in the pair-comparison of Panc-1 vs BxPC-3 and Panc-1 vs SW1990 implied that relatively high concentration of choline-like metabolites including phosphocholine (PC) and glycerolphosphocholine (GPC) may be significant metabolic features for poor differentiation of PDAC. According to previous study, the tumor-associated choline metabolism plays a key role in cell malignant transformation, tumor migration and metastasis [56, 57], characterized by elevated level of PC and total choline in tissue [45, 46]. Thereinto, the overexpression of choline kinase-α (Chk-a) induced by hypoxia-inducible factor (HIF) accounts for the increase of cellular PC and total choline [58], generating excessive phosphatidylcholine for biosynthesis of cell membrane. In addition, the EDI3-intermediated choline metabolism, a pathways verified in other solid tumor, can not only cleave GPC to form choline to supplement Kennedy pathway, but also generate glycerol-3-phosphate and its sequentially downstream intermediators for cellular signaling to regulate migration, invasion, proliferation and differentiation [57]. Other research detecting serum from PDAC patients also indicated that the choline metabolism were obviously altered and could potentially serve as biomarkers to detect PDAC in early stage. Thus, the difference of choline metabolism in PDAC could reflect and create a handful of regulatory functions on tumor progression and differentiation.

There are some pitfalls in this study. The PDAC models were established by using three PDAC cell lines which could only represent a part of metabonome landscape of pancreatic cancer, which inevitably lower the level of evidence provided from our study. The heterogeneity of pancreatic cancer in patients may compromise the directly clinical transformation application of our results. Thus, the further validation based on a large patient cohort will be performed in the future.

## Conclusions

In this study, we compared the serum metabonomic profiling between PDAC with different differentiations and successfully established pattern recognition models to distinguish with each other. The lower concentration of amino acids, glycolytic and glutaminolytic participators may serve as the predictors for poor differentiation of tumor. Thus, NMR-based metabonomic strategy can be a promising non-invasive approach to predict tumor differentiation preoperatively.

## Abbreviations

1-MH: 1-Methylhistidine
3-HB: 3-Hydroxybutyrate
AA: Acetoacetate
Ace: Acetate
Act: Acetone
Ala: Alanine
All: Allantoin
Bet: Betaine
Cho: Choline
Cit: Citrate
CPMG: Carr-Purcell-Meiboom-Gill
Cr: Creatine
EAAs: Essential amino acids
Eth: Ethanol
For: Formate
Fum: Fumarate
G: Glycerol
Gln: Glutamine
Glu: Glutamate
Gly: Glycine
GPC: Glycerolphosphocholine
His: Histidine
HOD: Residual water signal
IB: Isobutyrate
Ile: Isoleucine
L1: LDL
L2: VLDL
Lac: Lactate
LAT-1: L-type amino-acid transporter 1
Leu: Leucine
Lys: Lysine
Mal: Malonate
Met: Methionine
MG: Methylguanidine
m-I: myo-Inositol
Mol: Methanol
mTOR: Mammalian target-of-rapamycin
NAG: N-acetyl glycoprotein
NEAAs: Non-essential amino acids
NMR: Nuclear magnetic resonance
OPLS-DA: Orthogonal partial least squares discriminant analysis
PC: Phosphocholine
PCA: Principal component analysis
PDAC: Pancreatic ductal adenocarcinoma
Phe: Phenylalanine
PLS-DA: Partial least squares discriminant analysis
Py: Pyruvate
SGOC pathway: Serine-glycine-one-carbon pathway
Suc: Succinate
TCA: Tricarboxylic acid
Thr: Threonine
TMA: Trimethylamine
Trp: Tryptophan
Tyr: Tyrosine
Val: Valine
α-Glc: α-Glucose
β-Glc: β-Glucose
